# Fertility outcomes subsequent to medical and surgical treatment for ectopic pregnancy: A retrospective cohort study in Iran

**DOI:** 10.18502/ijrm.v19i10.9820

**Published:** 2021-11-04

**Authors:** Zahra Asgari, Venus Chegini, Reihaneh Hosseini, Mina Mohajeri, Iman Ansari

**Affiliations:** ^1^Department of Obstetrics and Gynecology, Arash Women's Hospital, Tehran University of Medical Sciences, Tehran, Iran.; ^2^Department of Obstetrics and Gynecology, Qazvin University of Medical Sciences, Qazvin, Iran.; ^3^Medical Students Research Committee, Shahed University, Tehran, Iran.

**Keywords:** Ectopic pregnancy, Fertility, Methotrexate, Salpingostomy, Salpingectomy.

## Abstract

**Background:**

Ectopic pregnancy (EP) and its treatment methods may affect subsequent fertility outcomes.

**Objective:**

To compare methotrexate (MTX), laparoscopic salpingostomy, and salpingectomy methods of EP treatment and their effects on fertility outcomes.

**Materials and Methods:**

This retrospective cohort study was performed on women receiving a definitive diagnosis of tubular EP from 2014 to 2017 at Arash Medical Center, Tehran, Iran. In total, 194 women were studied, of which 64 were treated with MTX, 52 underwent salpingostomy, and 78 underwent salpingectomy, depending on their clinical status. Basic information, obstetrics history, and major outcomes of the treatment after an 18-month follow-up, including recurrence of EP, miscarriage, and successful intrauterine pregnancy (IUP), were recorded and variables were compared among the three groups.

**Results:**

There was no significant difference in fertility outcomes among the three groups. Among the studied variables, predictors of successful IUP after EP treatment were multiparity (Hazard Ratio (HR): 1.37; 95%CI: 1.06-1.77), no history of miscarriage (HR: 2.37; 95%CI: 1.01-5.56), and a higher number of live births (HR: 1.54; 95%CI: 1.01-2.37). On the other hand, predictors of EP recurrence included nulliparity (HR: 1.61; 95%CI: 1.02-2.53) and a lower number of live births (HR: 3.84; 95%CI: 1.43-10.98). The effect of other factors, including the utilized therapeutic modalities, was not statistically significant.

**Conclusion:**

The current study results demonstrated that after an 18-month follow-up, fertility outcomes, including recurrence of EP and successful IUP, were not significantly different among the subjects with EP treated with MTX, salpingostomy, or salpingectomy. Further studies with long-term follow-ups are recommended.

## 1. Introduction

Ectopic pregnancy (EP) refers to the implantation of blastocysts outside the uterine endometrial cavity, which in > 90% of cases occurs in the fallopian tubes. EP is one of the most common causes of maternal mortality in the first trimester of pregnancy, causing approximately three-quarters of maternal deaths in the first trimester and one-tenth of maternal deaths in the whole of pregnancy (1). In developed countries, about 1-2% of pregnancies develop into EP, while in developing countries, its prevalence is up to 10 times higher (2). The prevalence of EP is increasing worldwide. The results of a study showed that the prevalence of EP in Iran during the past 15 yr was 3.7 per 1,000 pregnancies, almost twice as much as before 2005 (3); this increase was attributed to an increased prevalence of sexually transmitted diseases, increased tuboplasty following tubal ligation, emergency contraception, and the use of assisted reproductive techniques. The prevalence of EP in infertility centers has been estimated at 44-55 per 1,000 pregnancies (3, 4). Other EP risk factors include smoking, alcohol consumption of > 10 g/day, exposure to diethylstilbestrol, endometriosis, taking oral contraceptives before the age of 16, and a history of infertility or use of intrauterine contraceptive devices (5). EP risk factors may vary across cultures and races, but the risk factors of EP identified in Iranian women are a history of EP, abdominal-pelvic surgery, and nulliparity (6, 7). Complications of EP are highly significant and can be associated with high morbidity and mortality and affect fertility (2). There are various therapies for EP, the most common of which are: conservative treatment, medical treatment with methotrexate (MTX), and surgical treatments such as salpingostomy and salpingectomy (8). The utilized type of treatment depends on the subject's health status, location of EP, and serum level of beta human chorionic gonadotropin (β-hCG) (9, 10). Although some studies show that the outcomes of fertility are not significantly different among various treatments (11) and the years of successful fertility are almost identical across treatment modalities (12), others show that the utilized type of EP treatment affects the subsequent pregnancy outcomes. In some studies, conservative treatment (13), and in others MTX and salpingostomy, are preferred to other treatments to maintain fertility (14). Thus, the role of the type of treatment in maintaining fertility remains unclear (13).

Given the importance of this disease and its increasing prevalence and the necessity of finding the best treatment, the present study aimed to compare three methods to treat tubular EP (MTX, laparoscopic salpingostomy, and salpingectomy), and their effects on fertility outcomes.

## 2. Materials and Methods

### Study design 

This retrospective cohort study was performed on women receiving a definitive diagnosis of tubular EP from April 2014 to March 2017 at Arash Medical Center, which is affiliated with Tehran University of Medical Sciences, Tehran, Iran. Data were retrospectively collected from the hospital records using a non-probability sampling method.

### Participants

The inclusion criteria of the study were: women receiving an EP diagnosis based on serum β-hCG level and abdominal or vaginal ultrasound findings (15). Women receiving a non-tubular EP diagnosis, women undergoing both medical and surgical treatments, and women lost to follow-up or not providing the consent to participate in the study were excluded. The subjects were assigned to one of the three treatment groups according to their clinical status.

### Treatment groups

Women treated with MTX had stable hemodynamics, normal blood count, normal kidney and liver function, were not breastfeeding, had no severe and persistent pain, no pulmonary problems, no immunodeficiency, no active gastric ulcer, no active pelvic bleeding, had a serum β-hCG level < 5000 IU/L, EP with no fetal heartbeat, and a gestational sac diameter < 4 cm. In the single-dose regimen, 50 mg/m ${}^{2}$ of MTX was administered, and if the β-hCG level did not drop by > 15%, the second dose was administered on the fourth day; if the β-hCG level did not reduce by the seventh day, the third dose was also administered. On day 11, the β-hCG level was measured again; the surgical procedure was performed if the medical treatment failed. In the multi-dose regimen, 1 mg/kg of body weight was administered daily for up to four doses to reduce the β-hCG serum level by 15% or more. The hormone levels were measured on days one, three, five, and seven, and surgery was performed in the absence of the expected reduction of β-hCG level (16). In addition, women with a gestational sac diameter of > 4 cm or concomitant intrauterine pregnancy (IUP) underwent laparoscopic salpingostomy. Women with ruptured EP, unstable hemodynamics, and those who were not good candidates for salpingostomy underwent salpingectomy. Finally, out of the 194 studied women with tubular EP, 64 (33%) were treated with MTX, 52 (26.8%) underwent salpingostomy, and 78 (40.2%) underwent salpingectomy.

### Study outcomes

All subjects were instructed to use the most appropriate contraceptive method according to their conditions for three months after the treatment, and then they were advised to conceive. Routine outpatient follow-up was performed at the Obstetrics and Gynecology Clinic of the hospital. All women were followed up in October 2018, either in-person or by telephone, for the outcomes of treatment.

A checklist was designed and basic information was recorded, including age, body mass index, and history of EP, pelvic inflammatory disease, endometriosis and abdominal-pelvic surgery, as well as obstetrics history (including gravidity and parity, live birth, miscarriage, intrauterine fetal death), time of EP diagnosis and treatment, follow-up, and major outcomes of the treatment (including recurrence of EP, miscarriage, and successful IUP). Variables were compared among the three groups.

### Ethical considerations

The study was conducted in accordance with the Declaration of Helsinki, and the study protocol was approved by the Ethics Committee of Tehran University of Medical Sciences, Tehran, Iran (Code: IR.TUMS.MEDICINE.REC.1396.2931). Participants were assured of the confidentiality of their information and that the research results would be published anonymously. Signed consent forms were also obtained from the participants.

### Statistical analysis

Statistical Package for Social Science (SPSS) software, version 18 (IBM, Chicago, IL, USA) was employed for the statistical analysis. At first, the normal distribution of the data was assessed using the Kolmogorov-Smirnov test, and then quantitative and qualitative variables were expressed as mean ± standard deviation and frequency percentage, respectively. Chi-square and Fisher's exact tests were employed for statistical comparison of nominal variables between the groups. Independent sample *t *tests and analysis of variance or the Mann-Whitney and Kruskal-Wallis tests were employed to analyze the quantitative variables according to the distribution of samples in the statistical population. The univariate Cox regression model was also employed, and the results were expressed as a 95% confidence interval (CI) in terms of the hazard ratio. In addition, the probability of recurrent EP or IUP was estimated using the Kaplan-Meier estimator and log-rank test. In all statistical procedures, p < 0.05 was considered as the level of significance.

## 3. Results

The mean age of the subjects was 31.9 ± 5.6 yr, ranging from 18 to 50. There was no significant difference in terms of age among the treatment groups. In addition, there was no significant difference among the three groups in terms of body mass index or history of EP, cesarean section or underlying diseases (Table I).

The follow-up findings revealed that 104 individuals failed to conceive despite trying. Among the subjects that became pregnant, recurrent EP was observed in 16, miscarriage in 16, and successful IUP in 58 subjects. There was no significant difference in fertility outcomes among the three groups (Table II).

The effect of the independent variables and treatment on subsequent successful IUP was assessed using simple and multiple logistic regression models. Women were divided into two groups of successful and unsuccessful IUP. Based on the results of the Cox regression analysis in the current study, among the studied variables, the predictors of successful IUP after a medical treatment with MTX, salpingostomy, or salpingectomy were multiparity, no history of miscarriage, and a higher number of live births. On the other hand, predictors of EP recurrence included nulliparity and a lower number of live births. The effect of other factors, including the employed therapeutic modalities, was not statistically significant (Table III).

**Figure 1:**
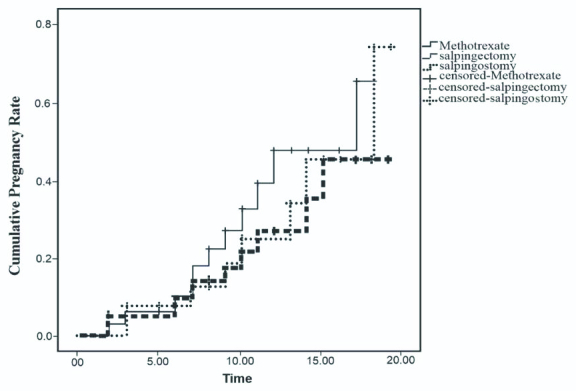
The Kaplan-Meier estimator showing the probability of successful IUP at 20 months after treatment with MTX, salpingostomy, or salpingectomy. The probability of successful IUP after the treatment was 34% with MTX, 26% with salpingostomy, and 31% with salpingectomy (log-rank: 0.86, p = 0.651).

**Figure 2:**
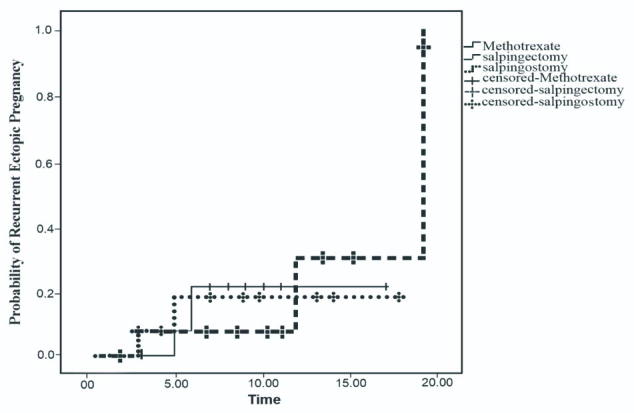
Kaplan-Meier estimator showing the probability of recurrence of EP at 20 months after treatment with MTX, salpingostomy, or salpingectomy. The risk of EP recurrence after the treatment with MTX, salpingostomy, and salpingectomy was 18, 20, and 15%, respectively (log-rank: 0.09, p = 0.956).

According to the Kaplan-Meier estimator, the probabilities of successful IUP and EP recurrence among the three treatment modalities were not significant (Figures 1 and 2).

**Table 1:** Basic characteristics in the three groups of study


	**Treatment group**			
	**MTX (n = 64)**	**Salpingectomy (n = 78)**	**Salpingostomy (n = 52)**	**Total (n = 194)**	**p-value**
Age (yr) ${}^{*}$	32.4 ± 6.2	32.2 ± 5.5	30.7 ± 4.9			31.9 ± 5.6	0.474
Body mass index (kg/m ${}^{2}$ ) ${}^{*}$	25.8 ± 3.9	24.9 ± 3.8	26.4 ± 4.8	25.6 ± 4.1	0.336
The subject history ${}^{**}$					
**Pelvic inflammatory disease**	8 (12.5)	0 (0)	6 (11.5)	14 (7.2)	0.062
**Endometriosis **	2 (3.1)	2 (2.6)	6 (11.5)	10 (5.1)	0.265
**Cesarean section**	20 (31.2)	26 (33.3)	14 (26.9)	60 (30.9)	0.86
**EP**	8 (12.5)	14 (17.9)	6 (11.5)	28 (14.4)	0.718
Obstetrics history ${}^{**}$					
**Live birth**	26 (40.6)	36 (46.1)	24 (46.1)	86 (44.3)	0.901
**Miscarriage**	34 (53.1)	26 (33.3)	26 (50)	86 (44.3)	0.202
**Intrauterine fetal death**	0 (0)	4 (5.1)	2 (3.8)	6 (3.1)	0.619
**Time of EP diagnosis** ${}^{*}$	6.1 ± 1.4	6.0 ± 2.2	6.5 ± 1.9	6.2 ± 1.9	0.537
Data are expressed as *Mean ± SD (Analysis of Variance), ${}^{**}$ Frequency percentage (Chi-square test), MTX: Methotrexate, EP: Ectopic pregnancy					

**Table 2:** Follow-up results based on the three study groups


	**Treatment group**			
	**MTX (n = 64)**	**Salpingectomy (n = 78)**	**Salpingostomy (n = 52)**	**Total (n = 194)**	**p-value**
**No pregnancy**	30 (46.9)	48 (61.5)	26 (50)	104 (53.7)	0.452
**Recurrent EP**	6 (9.4)	6 (7.7)	4 (7.7)	16 (8.2)	0.961
**Miscarriage**	6 (9.4)	4 (5.1)	6 (11.5)	16 (8.2)	0.629
**IUP**	22 (34.3)	20 (25.7)	16 (30.8)	58 (29.9)	0.725
Data are expressed as frequency percentage, Chi-square test, MTX: Methotrexate, EP: Ectopic pregnancy, IUP: Intrauterine pregnancy					

**Table 3:** Cox proportional hazards model for predicting IUP and recurrence of EP after medical treatment with MTX or surgical treatment with salpingostomy or salpingectomy


	**IUP**		**Recurrence of EP**	
	**Hazard ratio (95% CI)**	**p-value**	**Hazard ratio (95% CI)**	**p-value**
**Age (yr)**	0.99 (1.06 -0.93)	0.483	1.04 (0.93-1.16)	0.483
**Body mass index (kg/m ${}^{2}$ )**	0.97 (0.89-1.07)	0.831	1.02 (0.85-1.24)	0.831
Pelvic inflammatory disease ${}^{*}$	0.83 (0.11-6.12)	0.853	0.047 (0.0-NE)	0.754
Endometriosis ${}^{*}$	0.93 (0.22-3.95)	0.992	0.045 (0.0-NE)	0.653
Cesarean section ${}^{*}$	2.07 (0.99-4.32)	0.052	1.56 (0.35-6.99)	0.564
EP ${}^{*}$	0.7 (0.21-2.30)	0.535	2.89 (0.55-15.13)	0.208
**Parity**	1.37 (1.06-1.77)	0.015	1.61 (1.02-2.53)	0.039
**Live birth**	1.54 (1.01-2.37)	0.049	3.84 (1.43-10.98)	0.012
Miscarriage ${}^{*}$	2.37 (1.01-5.56)	0.047	0.30 (0.06-1.53)	0.147
**Therapeutic modality**				
**Salpingectomy ${}^{\#}$ **	0.67 (0.28-1.56)	0.363	0.76 (0.13-4.59)	0.886
**Salpingostomy ${}^{\#}$ **	0.81 (0.32-2.03)	0.653	0.92 (0.15-5.54)	0.452
${}^{*}$ Reference is the subject without a history. ${}^{\#}$ Reference is the subject treated with MTX. EP: Ectopic pregnancy, IUP: Intrauterine pregnancy, CI: Confidence interval, NE: Not estimable				

## 4. Discussion

Two issues are always considered before selecting the treatment regimen for tubal EP: the impact of salpingectomy on future fertility and the risk of recurrent EP after conservative treatment (15). Unfortunately, to the best of the authors' knowledge, there is still no consensus on EP treatment, and most studies recommend further investigations comparing the impact of EP therapies on major fertility outcomes, including successful IUP and recurrent EP. Therefore, this retrospective cohort study aimed to compare the outcomes of medical and surgical treatment of tubal EP from 2014 to 2017.

The results of this study demonstrated 8% EP recurrence and 30% successful IUP during an 18-month follow-up, while there were no statistically significant differences in fertility outcomes, including EP recurrence, successful IUP or abortion, among the three treatment modalities. Also, no significant difference was noted in the probability of successful IUP or EP recurrence after the treatment based on the Kaplan-Meier estimator results.

Other studies from around the world have compared EP therapies. In this regard, the results of a study in Iran indicated no differences in fertility outcomes between the two methods of single-dose MTX and laparoscopic salpingectomy, which is consistent with the findings of the present study (17). Researchers in Poland and in Germany found that fertility outcomes did not differ significantly across the three treatment modalities, which is again consistent with the findings of this study (12, 18). However, contrary to the results of this study, in France was reported that conservative treatment with MTX was significantly superior to the surgical procedure, and it was associated with more favorable pregnancy outcomes (13). The geographical, cultural, and demographic differences, as well as the follow-up time, may have affected the results of the present and mentioned studies. The results of a study in Italy showed that in salpingostomy and salpingectomy groups, IUP rates were 56.1% and 60%, respectively and EP recurrence rates were 5.3% and 18.7%, respectively; in addition, the IUP rate was higher in both treatment groups than in the present study (19). The mean follow-up time in their study was 24 months, which may justify the differences in the results of the two studies, but no significant differences were found between the surgical groups in either of the two studies in terms of fertility outcomes. However, it seems that the risk of recurrent EP in women treated with salpingectomy may be lower than that of salpingostomy over time. Some researchers in Saudi Arabia, after a 5-yr follow-up of women with EP, reported that salpingectomy in comparison with salpingostomy, and laparoscopy in comparison with laparotomy, were associated with a lower risk of EP recurrence (20). On the other hand, a study in Turkey, consistent with the results of the present study, found no significant differences between treatment modalities in terms of therapeutic outcomes; they concluded that women undergoing salpingectomy may conceive later in life compared with the ones in the other groups (21). Results of the present study and other studies have shown that the outcomes of salpingectomy may vary over time (17-21).

According to the results of the Cox regression model in the current study, the predictors of IUP were no history of miscarriage and a higher number of live births, and the predictor of EP recurrence was a lower number of live births. However, the effect of other studied factors, including the therapeutic modalities used, was not statistically significant. In this regard, in China was suggested that multiparity was associated with a lower risk of recurrent EP, which is consistent with the results of the present study (22). Also consistent with our findings, the results of a study showed that therapeutic modalities were not independent risk factors for EP recurrence (12). Furthermore, it was reported that childbearing history was a predictive factor for EP recurrence (13). Although no significant differences were found in terms of fertility outcomes between the treatment groups, which is consistent with the results of the present study, a higher age of women was noted as a risk factor for infertility (23). This difference may be attributed to the impact of age on fertility that was also observed regarding successful IUP and EP recurrence in the current study.

Based on the results of our study and similar studies in this field, it seems that there is no significant difference in fertility outcomes among medical and surgical therapies for tubular EP. Therefore, the clinical condition of the subjects, as well as other factors such as cost-efficacy, should be considered in choosing treatment modalities. In this regard, in Tunisia it was reported that the medical treatment with MTX was costlier due to a longer hospital stay (24), although another study revealed that medical treatment with MTX may shorten hospital stay and reduce treatment costs (25).

One of the limitations of the current study was the short-term follow-up of the subjects. Since fertility outcomes may change over time, further studies are recommended with longer follow-up durations. In addition, due to different pregnancy outcomes following various EP therapies worldwide, systematic reviews and meta-analyses in this field are necessary.

## 5. Conclusion

The results of the current study demonstrated that with an 18-month follow-up, fertility outcomes, including recurrence of EP and successful IUP, were not significantly different among women with EP treated with MTX, salpingostomy, or salpingectomy. Further studies with longer-term follow-ups are recommended.

##  Conflict of Interest

The authors declare no conflict of interest.

## References

[B1] GBD 2015 Maternal Mortality Collaborators (2016). Global, regional, and national levels of maternal mortality, 1990-2015: A systematic analysis for the Global Burden of Disease Study 2015. Lancet.

[B2] Gaskins AJ, Missmer SA, Rich-Edwards JW, Williams PL, Souter I, Chavarro JE (2018). Demographic, lifestyle, and reproductive risk factors for ectopic pregnancy. Fertil Steril.

[B3] Hasani M, Keramat A, Khosravi A, Oshrieh Z, Hasani M (2016). [Prevalence of ectopic pregnancy in Iran: A systematic review and meta-analysis]. Iran J Obstet Gynecol Infertil.

[B4] Mokhtari Zanjani P, Ahmadnia E, Kharaghani R (2019). Ectopic pregnancy rate in Iranian midwifery clients and infertile patients treated by assisted reproductive technologies. J Evid Based Med.

[B5] Yong PJ, Matwani S, Brace Ch, Quaiattini A, Bedaiwy MA, Albert A, et al (2020). Endometriosis and ectopic pregnancy: A meta-analysis. J Minim Invasive Gynecol.

[B6] Bouzari Z, Yazdani Sh, Alizadeh M, Ghanbarpour A, Bijani A, Lakaei F (2019). The risk factors for cctopic pregnancy. J Babol Univ Med Sci.

[B7] Parashi S, Moukhah S, Ashrafi M (2014). Main risk factors for ectopic pregnancy: A case-control study in a sample of Iranian women. Int J Fertil Steril.

[B8] Ting WH, Lin HH, Hsiao SM (2019). Factors predicting persistent ectopic pregnancy after laparoscopic salpingostomy or salpingotomy for tubal pregnancy: A retrospective cohort study. J Minim Invasive Gynecol.

[B9] Kirk E, Bottomley C, Bourne T (2014). Diagnosing ectopic pregnancy and current concepts in the management of pregnancy of unknown location. Hum Reprod Update.

[B10] Pakniat H, Bahman A, Ansari I (2019). The relationship of pregnancy-associated plasma protein A and human chorionic gonadotropin with adverse pregnancy outcomes: A prospective study. J Obstet Gynaecol India.

[B11] Helmy S, Sawyer E, Ofili-Yebovi D, Yazbek J, Ben Nagi J, Jurkovic D (2007). Fertility outcomes following expectant management of tubal ectopic pregnancy. Ultrasound Obstet Gynecol.

[B12] Talarczyk-Desole J, WrÃ³bel M, Niepsuj-BiniaÅ› J, Szymanowski K, Opala T, Pawelczyk L, et al

[B13] de Bennetot M, Rabischong B, Aublet-Cuvelier B, Belard F, Fernandez H, Bouyer J, et al (2012). Fertility after tubal ectopic pregnancy: Results of a population-based study. Fertil Steril.

[B14] Beall S, DeCherney AH (2012). Management of tubal ectopic pregnancy: Methotrexate and salpingostomy are preferred to preserve fertility. Fertil Steril.

[B15] Carusi D (2019). Pregnancy of unknown location: Evaluation and management. Semin Perinatol.

[B16] Inal ZO, Inal HA (2018). Comparison of four methods of treating ectopic pregnancy: A retrospective cohort study. Geburtshilfe Frauenheilkd.

[B17] Yousefnezhad A, Pirdehghan A, Roshandel Rad M, Eskandari A, Ahmadi S (2018). Comparison of the pregnancy outcomes between the medical and surgical treatments in tubal ectopi pregnancy. Int J Reprod Biomed.

[B18] Lermann J, Segl P, Jud SM, Beckmann MW, Oppelt P, Thiel FC, et al (2014). Low-dose methotrexate treatment in ectopic pregnancy: A retrospective analysis of 164 ectopic pregnancies treated between 2000 and 2008. Arch Gynecol Obstet.

[B19] Lagana AS, Vitale SG, De Dominici R, Padula F, Rapisarda AM, Biondi A, et al (2016). Fertility outcome after laparoscopic salpingostomy or salpingectomy for tubal ectopic pregnancy: A 12-years retrospective cohort study. Ann Ital Chir.

[B20] Ellaithy M, Asiri M, Rateb A, Altraigey A, Abdallah K (2018). Prediction of recurrent ectopic pregnancy: A five-year follow-up cohort study. Eur J Obstet Gynecol Reprod Biol.

[B21] Turan V (2011). Fertility outcomes subsequent to treatment of tubal ectopic pregnancy in younger Turkish women. J Pediatr Adolesc Gynecol.

[B22] Zhang D, Shi W, Li C, Yuan JJ, Xia W, Xue RH, et al

[B23] Jamard A, Turck M, Pham AD, Dreyfus M, Benoist G (2016). [Fertility and risk of recurrence after surgical treatment of an ectopic pregnancy (EP): Salpingostomy versus salpingectomy]. J Gynecol Obstet Biol Reprod (Paris).

[B24] Fadhlaoui A, Oueslati H, Khedhiri Z, Khrouf M, Chaker A, Zhioua F (2013). [Cost of medical treatment with methotrexate for ectopic pregnancy. Study comparing medical treatment versus laparoscopy: Experience of Aziza Othmana Hospital] Tunis Med.

[B25] Ansong E, Illahi GS, Shen L, Wu X (2019). Analyzing the clinical significance of postoperative methotrexate in the management of early abdominal pregnancy: Analysis of 10 cases. Ginekol Pol.

